# Using Land Cover Data to Characterize Living Environments of Geocoded Addresses: Estes et al. Respond

**DOI:** 10.1289/ehp.0901863R

**Published:** 2010-03

**Authors:** Maurice G. Estes, Mohammad Z. Al-Hamdan, William Crosson, Sue M. Estes, Dale Quattrochi, Shia Kent, Leslie Ain McClure

**Affiliations:** Universities Space Research Association, NASA–Marshall Space Flight Center, National Space Science and Technology Center, Huntsville, Alabama, E-mail: maury.g.estes@nasa.gov; Earth Science Office, NASA–Marshall Space Flight Center, National Space Science and Technology Center, Huntsville, Alabama; Department of Biostatistics, University of Alabama–Birmingham, Birmingham, Alabama

We appreciate the insightful and informative letter about the methodology used in our article (Estes et al. 2009). We agree with Zandbergen about the methodology employed by the SAS/GIS software used for geocoding the REGARDS (REasons for Geographic and Racial Differences in Stroke)participants. As one of the REGARDS study goals, we plan to re-geocode the participants using a more accurate method. However, because our article focused on classifying the “living environment” (defined as urban, suburban, and rural) and because most people do not spend the majority of their time at their house or within the raw resolution area (30 m × 30 m), the geocoding errors that are in the levels of tens of meters become less relevant. This is true especially when we resample to a coarser resolution (1 km vs. 30 m), as we did in our methodology to characterize the participants’ living environment.

With respect to the misclassification that may be introduced due to the resolution used to classify participants, Zandbergen is correct that resampling to different resolutions did change the classification of the participants. However, the results of the analyses were consistent regardless of the resolution of the classification, indicating that while this may influence the exposure itself, it does not influence the relationship between the exposure and the outcome.
